# Investigation of Multiple Susceptibility Loci for Inflammatory Bowel Disease in an Italian Cohort of Patients

**DOI:** 10.1371/journal.pone.0022688

**Published:** 2011-07-27

**Authors:** Anna Latiano, Orazio Palmieri, Tiziana Latiano, Giuseppe Corritore, Fabrizio Bossa, Giuseppina Martino, Giuseppe Biscaglia, Daniela Scimeca, Maria Rosa Valvano, Maria Pastore, Antonio Marseglia, Renata D'Incà, Angelo Andriulli, Vito Annese

**Affiliations:** 1 Gastroenterology Unit, IRCCS “Casa Sollievo della Sofferenza,” San Giovanni Rotondo, Italy; 2 Division of Paediatrics, IRCCS “Casa Sollievo della Sofferenza,” San Giovanni Rotondo, Italy; 3 Department of Surgical and Gastroenterological Sciences, University of Padua, Padua, Italy; 4 Division of Gastroenterology, University Hospital Careggi, Florence, Italy; Ulm University, Germany

## Abstract

**Background:**

Recent GWAs and meta-analyses have outlined about 100 susceptibility genes/loci for inflammatory bowel diseases (IBD). In this study we aimed to investigate the influence of SNPs tagging the genes/loci *PTGER4*, *TNFSF15*, *NKX2-3*, *ZNF365*, *IFNG*, *PTPN2*, *PSMG1*, and *HLA* in a large pediatric- and adult-onset IBD Italian cohort.

**Methods:**

Eight SNPs were assessed in 1,070 Crohn's disease (CD), 1,213 ulcerative colitis (UC), 557 of whom being diagnosed at the age of ≤16 years, and 789 healthy controls. Correlations with sub-phenotypes and major variants of *NOD2* gene were investigated.

**Results:**

The SNPs tagging the *TNFSF15*, *NKX2-3*, *ZNF365*, and *PTPN2* genes were associated with CD (*P* values ranging from 0.037 to 7×10^−6^). The SNPs tagging the *PTGER4*, *NKX2-3*, *ZNF365*, *IFNG, PSMG1*, and *HLA* area were associated with UC (*P* values 0.047 to 4×10^−5^). In the pediatric cohort the associations of *TNFSF15, NKX2-3* with CD, and *PTGER4, NKX2-3, ZNF365, IFNG, PSMG1* with UC, were confirmed. Association with *TNFSF15* and pediatric UC was also reported. A correlation with *NKX2-3* and need for surgery (*P*  =  0.038), and with *HLA* and steroid-responsiveness (*P*  =  0.024) in UC patients was observed. Moreover, significant association in our CD cohort with *TNFSF15* SNP and colonic involvement (P  =  0.021), and with *ZNF365* and ileal location (*P*  =  0.024) was demonstrated.

**Conclusions:**

We confirmed in a large Italian cohort the associations with CD and UC of newly identified genes, both in adult and pediatric cohort of patients, with some influence on sub-phenotypes.

## Introduction

The pathogenesis of inflammatory bowel diseases (IBD), namely Crohn's disease (CD) and ulcerative colitis (UC), is still incompletely understood, but it is widely accepted that the two conditions result from an inappropriate and exaggerated mucosal immune response to constituents of the intestinal flora in a genetically susceptible host [1], [2]. In the past year, genome-wide association (GWA) studies have identified several genes involved in the pathogenesis of IBD and, subsequently GWAS meta-analysis has led to confirmation of more than 70 genes or loci that confer susceptibility to CD and 47 to UC, mostly in adult populations [3], [4], [5]. In particular, GWA and replications studies identified gene variants, including protein tyrosine phosphathase nonreceptor type 2 (*PTPN2*), NK2 transcription factor related locus 3 (*NKX2-3*), and tumor necrosis factor superfamily member 15 (*TNFSF15*) [6], [7], [8]. A subsequent North American GWA study identified two additional loci for UC located on chromosome 1p36 and 12q15, each of them harboring multiple genes, including several with a definite role in inflammation and immunity, like group II secreted phospholipase A2 (*PLA2G2E*), interferon gamma (*IFNG*), interleukin 26 (*IL26*) and interleukin 22 (*IL22*). In addition, combined genome-wide significant evidence for association was found at two additional loci, namely HLA on chromosome 6p21 and *IL23R* (interleukin-23 receptor) on chromosome 1p31 [9]. Finally, a GWA study carried out in pediatric-onset IBD patients identified two novel IBD loci located on chromosome 20q13 and 21q22, close to the tumor necrosis factor receptor superfamily member 6B (*TNFRSF6B*) and proteasome-assembling chaperone 1 (*PSMG1*) genes [10].

In the present study, we investigated whether potential loci reported in the meta-analysis and GWA studies on chromosomes 5p13 (*PTGER4*), 12q15 (*IFNG, IL22, IL26*), 6p21 (*HLA*), 21q22 (*PSMG1*), and *PTPN2*, *NKX2-3*, *TNFSF15*, were associated in a large and phenotypically well-characterized Italian cohort of IBD patients, and we also attempted to elucidate their involvement in early onset disease. In addition, we tested the potential epistasis between these variants and IBD-associated *NOD2/CARD15* SNPs, as well as possible genotype-phenotype correlations.

## Materials and Methods

### Ethics statement

The IBD cohort and unaffected controls were recruited from adult individuals referred the IRCCS, “Casa Sollievo della Sofferenza “ Hospital in San Giovanni Rotondo, and from pediatric centers of the Italian Society of Pediatric Gastroenterology, Hepatology and Nutrition (SIGENP). Written informed consent was obtained from all adult participants and, for patients under age of 19 years, from related parents. Ethical approval was acquired from the Ethics Review Board of “Casa Sollievo della Sofferenza” Hospital, San Giovanni Rotondo, and each participating center approved the recruitment protocol. The study was supported by a grant from the Italian Minister of the Health (RC0902GA33).

Extensive clinical characterization was available for all patients. The diagnosis of CD or UC was established by conventional clinical, radiological, endoscopic, and histological findings [11]. The CD phenotype was classified based on age at disease onset (A), maximal extent of disease (L), and behavior (B) according to the Montreal classification [12], [13]. In patients with UC the colon location was also classified according to the Montreal classification, by distinguishing ulcerative proctitis (E1), left-side colitis (E2), and extensive colitis (E3). For all IBD patients further clinical characteristics were analyzed, namely the occurrence of previous resective surgery, IBD family history, smoking habits, extra-intestinal manifestations and response to medical therapy. More specifically the need for use of corticosteroids, immunosuppressors (thiopurines and methotrexate) and anti-TNF agents were evaluated. In addition, on the basis of review of medical records, patients with the use of corticosteroid (CS) were classified as CS-responder (at least one course of systemic steroids with clinical remission reported in the medical history), or CS-refractory (when an unsuccessful clinical response was achieved leading to alternative therapies like surgery, use of anti-TNF or other immunosuppressors drugs) [14], [15]. Patients with incomplete or unclear information were excluded from this analysis.

### SNPs analysis and genotyping

We selected 8 polymorphisms for genotyping: three of them (rs4613763, rs4263839, rs11190140) identified by the CD GWA study meta-analysis [3], two (rs10761659, rs2542151) by the WTCCC [6] as showing the strongest association signals, two (rs2395185, rs1558744) by the first UC GWA study [9], and one (rs2836878) by a GWA analysis for early-onset IBD [10]. The genotypic variants in the *NOD2*/*CARD15* gene (rs2066844, rs2066845, rs2066847) had already been analyzed for all patients and controls. Genomic DNA was extracted from peripheral blood leukocytes by standard procedures using the DNA blood maxi kit from Qiagen (Hilden, Germany) in accordance with the manufacturer's instructions. All of the genotyping was performed at the Molecular Laboratory of the Gastroenterology Unit at the San Giovanni Rotondo Hospital, Italy. Genotyping was performed using Custom Taqman® SNP assay (Applied Biosystems, Foster City, CA), following manufacturer's instructions. The overall success rate of the genotyping assay was over 98%.

### Statistical analysis

Statistical analysis was performed using Haploview Software version 4.1 (http://www.broad.mit.edu/personal/mpg/haploview) and SPSS software version 13.0 (Chicago, IL, USA). Hardy-Weinberg Equilibrium (HWE) tests were performed for all investigated polymorphisms independently among cases and controls. For the case-control analysis, comparisons of genotypes and allele frequencies was performed using *Χ* ^2^ or Fisher's exact test, where appropriate. Genotype-phenotype associations were first analyzed by means of univariate analysis and subsequently expressed as Odds Ratios (OR) with 95% confidence intervals (95% CI) by means of stepwise logistic regression analysis. For detecting gene-gene interactions we used a logistic regression based on forward stepwise selection procedures using the number of risk alleles as predictor variable. P values of less than 0.05 were considered significant.

## Results

### Case-control analysis with IBD patients

A total of 2283 individuals with IBD, including 1070 with CD and 1213 with UC, was analyzed. The control group consisted of 789 individuals from the same ethnicity who did not have IBD (neither CD nor UC). Clinical and demographic features of the IBD cases are listed in **Table 1**. There were 296 CD patients and 261 UC patients with the initial diagnosis before their 16^th^ birthday. The adult cohort was constituted of 774 patients with CD and 952 with UC.

**Table 1:** Clinical and demographic characteristics of the study population.

	CD	UC
	n = 1070	n = 1213
***Gender***		
F	468 (44)	511 (42)
M	602 (56)	702 (58)
***Duration of follow-up (yr)***		
mean ± SD	8±7	9±7
median (range)	6 (1–37)	7 (1–41)
***Age at diagnosis (yr)***		
mean ± SD	27±15	31±16
median (range)	25 (1–79)	28 (1–83)
≤16 (A1)	296 (28)	261 (21)
17–40 (A2)	586 (55)	642 (53)
> 40 (A3)	188 (17)	310 (26)
***Disease location CD, n (%)***		
Ileum (L1±L4)	331 (31)	
Colon (L2±L4)	265 (25)	
leo-colon (L3±L4)	465 (43)	
Upper GI (L4)	9 (1)	
***Disease extent UC, n (%)***		
Rectum (E1)		139 (11)
Left colon (E2)		554 (46)
Pancolitis (E3)		520 (43)
***Disease behavior CD, n (%)***		
Inflammatory	677 (63)	
Stricturing	279 (26)	
Penetrating	114 (11)	
***Perianal Disease***		
No	882 (82)	
yes	188 (18)	
***EIM***		
No	651 (61)	908 (75)
yes	419 (39)	305 (25)
***Family history***		
No	959 (90)	1107 (91)
yes	111 (10)	106 (9)
* ***Smoker***		
Yes	292 (32)	164 (15)
No	490 (53)	622 (57)
Ex	140 (15)	311 (28)
***Resection***		
No	749 (70)	1082 (89)
yes	321 (30)	131 (11)
***Steroids***		
Refractory	83 (13)	101(13)
Responder	569 (87)	673 (87)
**IMS (AZT/6MP, Ciclo, MTX)**		
No	676 (42)	927(58)
Yes	394 (58)	286 (42)
**Infliximab**		
No	914 (85)	1168 (96)
Yes	156 (15)	45 (4)

CD: Crohn's disease; UC: ulcerative colitis; EIM: extra-intestinal manifestations.

IMS: immunosuppressors.

*For 148 CD and 116 UC patients the information is missing.

We compared single-marker allele frequencies using χ^2^ statistics (**Tables 2** **–** **3**). Frequencies were in accordance with the Hardy-Weinberg equilibrium. In the overall cohort of IBD patients, four markers were above the threshold for significance for CD: the *TNFSF15* (rs4263839, P  =  7.183×10^−6^), *NKX2-3* (rs11190140, P  =  0.003), *ZNF365* (rs10761659, P  =  0.007), and *PTPN2* (rs2542151, P  =  0.037). Of these four markers, two, namely, rs11190140 (P  =  4×10^−5^), and rs10761659 (P  =  4×10^−5^), were also associated with UC. In this latter subset of patients, four more markers, *PTGER4* rs4613763 (P  =  0.012), *HLA*-*BTNL2* rs2395185 (P  =  0.001), *IFNG-IL22-IL26* rs1558744 (P  =  0.007), and *PSMG1* rs2836878 (P  =  0.047) were exclusively associated.

**Table 2:** Genotype distribution of associated SNPs in Crohn's disease (CD) patients and healthy controls.

		No. of CD genotyped			No. of control genotyped				
Gene(s) or locus	SNP	AA*	Aa	aa	AA*	Aa	aa	P value	OR (95% CI)
*PTGER4*	rs4613763	7 (1.1%)	127 (19.3%)	523 (79.6%)	2 (0.4%)	86 (15.7%)	460 (83.9%)	0.0531	1.34 (0.99–1.80)
*HLA, BTNL2*	rs2395185	-	-	-	22 (5.1%)	154 (35.7%)	256 (59.2%)	-	-
*TNFSF15*	rs4263839	57 (6.3%)	322 (35.2%)	535 (58.5%)	74 (9.4%)	339 (43.0%)	376 (47.6%)	7.183E-06	0.65 (0.53–0.78)
*NKX2-3*	rs11190140	163 (18.8%)	437 (50.2%)	270 (31.0%)	176 (22.5%)	414 (52.9%)	193 (24.6%)	0.0039	0.73 (0.58–0.90)
*ZNF365*	rs10761659	240 (30.2%)	379 (47.7%)	175 (22.1%)	131 (24.3%)	255 (47.2%)	154 (28.5%)	0.0070	1.41 (1.09–1.81)
*IFNG, IL22, IL26*	rs1558744	-	-	-	77 (13.9%)	262 (47.4%)	214 (38.7%)	-	-
*PTPN2*	rs2542151	21 (2.4%)	229 (26.3%)	622 (71.3%)	10 (1.3%)	178 (22.8%)	591 (75.9%)	0.0371	1.26 (1.01-1.57)
*PSMG1*	rs2836878	10 (3.4%)	113 (38.3%)	172 (58.3%)	43 (7.6%)	203 (35.9%)	320 (56.5%)	ns	ns

A* denotes a risk allele.

OR: odds ratio; CI: confidence interval.

SNP: single nucleotide polymorphism.

**Table 3:** Genotype distribution of associated SNPs in ulcerative colitis (UC) patients and healthy controls.

		No. of UC genotyped			No. of control genotyped				
Gene(s) or locus	SNP	AA*	Aa	aa	AA*	Aa	aa	P value	OR (95% CI)
*PTGER4*	rs4613763	8 (1.2%)	142 (20.5%)	542 (78.3%)	2 (0.4%)	86 (15.7%)	460 (83.9%)	0.0126	1.45 (1.08–1.93)
*HLA, BTNL2*	rs2395185	29 (3.7%)	221 (28.1%)	537 (68.2%)	22 (5.1%)	154 (35.7%)	256 (59.2%)	0.0017	0.68 (0.53–0.86)
*TNFSF15*	rs4263839	66. (7.3%)	375 (41.6%)	460 (51.1%)	74 (9.4%)	339 (43.0%)	376 (47.6%)	ns	ns
*NKX2-3*	rs11190140	162 (17.9%)	438 (48.3%)	306 (33.8%)	176 (22.5%)	414 (52.9%)	193 (24.6%)	4E-05	0.64 (0.52–0.79)
*ZNF365*	rs10761659	168 (28.8%)	309 (53.0%)	106 (18.2%)	131 (24.3%)	255 (47.2%)	154 (28.5%)	4E-05	1.79 (1.36–2.38)
*IFNG, IL22, IL26*	rs1558744	167 (20.1%)	401 (48.2%)	264 (31.7%)	77 (13.9%)	262 (47.4%)	214 (38.7%)	0.0076	1.36 (1.08–1.70)
*PTPN2*	rs2542151	10 (1.1%)	232 (25.4%)	672 (73.5%)	10 (1.3%)	178 (22.8%)	591 (75.9%)	ns	ns
*PSMG1*	rs2836878	32 (3.7%)	302 (34.5%)	540 (61.8%)	43 (7.6%)	203 (35.9%)	320 (56.5%)	0.0473	0.80 (0.65–0.99)

A* denotes a risk allele.

OR: odds ratio; CI: confidence interval.

SNP: single nucleotide polymorphism.

To determine whether the previously identified variants were shared by both adult and pediatric individuals, we stratified all IBD patients according to their age at initial diagnosis. For the adult subset, associations were confirmed for all SNPs for either CD and UC with two remarkable exceptions: the rs4613763 variant, which was associated with CD (P  =  0.031)(**Table 4**), and the rs2836878 variant which lost the association for UC (**Table 5**). Concerning childhood-onset cohort, the genotype frequencies of all considered polymorphisms remained significantly associated for UC, with the exception of the rs2395185 variant (**Table 5**). In CD pediatric patients the association was confirmed for rs4263839 (P  =  0.008) and rs11190140 (P  =  0.007) variants (**Table 4**).

**Table 4:** Genotype distribution of associated SNPs in adult- and childhood-onset Crohn's disease (CD) cohort.

	No. of Adult CD genotyped			No. of Pediatric CD genotyped					
SNP	AA	Aa	aa	AA	Aa	aa		*P* value	OR (95% CI)
rs4613763	6 (1.2%)	99 (20.0%)	389 (78.8%)	1 (0.6%)	28 (17.2%)	134 (82.2%)	***Adult*** ***Pediatric***	0.0310ns	1.41 (1.03–1.93)ns
rs2395185	-	-	-	-	-	-	***Adult*** ***Pediatric***	--	--
rs4263839	35 (5.5%)	223 (35.2%)	376 (59.3%)	22 (7.8%)	99 (35.4%)	159 (56.8%)	***Adult*** ***Pediatric***	<0.0010.0087	0.62 (0.51–0.77)0.69 (0.52–0.91)
rs11190140	116 (19.4%)	301 (50.4%)	180 (30.2%)	47 (17.2%)	136 (49.8%)	90 (33.0%)	***Adult*** ***Pediatric***	0.02580.0075	0.84 (0.72–0.98)0.66 (0.49–0.89)
rs10761659	187 (30.4%)	301 (48.8%)	128 (20.8%)	53 (29.8%)	78 (43.8%)	47 (26.4%)	***Adult*** ***Pediatric***	0.0022ns	1.52 (1.16–1.99)ns
rs1558744	-	-	-	-	-	-	***Adult*** ***Pediatric***	--	--
rs2542151	13 (2.1%)	169 (27.8%)	426 (70.1%)	8 (3.0%)	60 (22.8%)	196 (74.2%)	***Adult*** ***Pediatric***	0.0153ns	1.34 (1.06–1.70)ns
rs2836878	-	-	-	-	-	-	***Adult*** ***Pediatric***	--	--

**Table 5:** Genotype distribution of associated SNPs in adult- and childhood-onset ulcerative colitis (UC) cohort.

	No. of Adult UC genotyped			No. of Pediatric UC genotyped					
SNP	AA	Aa	aa	AA	Aa	aa		*P* value	OR (95% CI)
rs4613763	5 (5.2%)	78 (18.4%)	341 (80.42%)	3 (1.1%)	64 (23.9%)	201 (75.0%)	***Adult*** ***Pediatric***	ns0.0022	ns1.74 (1.21–2.49)
rs2395185	19 (3.1%)	176 (28.4%)	425 (68.5%)	10 (6.0%)	45 (26.9%)	112 (67.1%)	***Adult*** ***Pediatric***	0.0019ns	0.67 (0.52–0.86)ns
rs4263839	50 (7.5%)	289 (43.4%)	327 (49.1%)	16 (6.8%)	86(36.6%)	133 (56.6%)	***Adult*** ***Pediatric***	ns0.0161	ns0.69 (0.52–0.94)
rs11190140	132 (19.8%)	313 (46.9%)	222 (33.3%)	30 (12.6%)	125 (52.3%)	84 (35.1%)	***Adult*** ***Pediatric***	0.00030.0014	0.66 (0.52–0.82)0.60 (0.44–0.82)
rs10761659	121 (27.7%)	235 (53.8%)	81 (18.5%)	47 (32.2%)	74 (50.7%)	25 (17.1%)	***Adult*** ***Pediatric***	0.00030.0054	1.75 (1.29–2.38)1.93 (1.21–3.09)
rs1558744	127 (18.7%)	329 (48.5%)	223 (32.8%)	40 (26.1%)	72 (47.1%)	41 (26.8%)	***Adult*** ***Pediatric***	0.03260.0067	1.29 (1.02–1.63)1.72 (1.16–2.56)
rs2542151	8 (1.2%)	169 (24.9%)	500 (73.9%)	2 (0.8%)	63 (26.6%)	172 (72.6%)	***Adult*** ***Pediatric***	nsns	nsns
rs2836878	30 (4.2%)	252 (35.6%)	426 (60.2%)	2 (1.2%)	50 (30.1%)	114 (68.7%)	***Adult*** ***Pediatric***	ns0.0051	ns0.59 (0.41–0.86)

### Genotype association with IBD phenotypes

We also assessed whether the investigated SNPs could bear an influence on specific disease phenotypes, such as gender, disease location and behavior, resective surgery, family history, smoking habit, extra-intestinal manifestations, and medical therapies. At logistic regression analysis (**Table 6**), using a custom/stepwise model (forward entry) and after correction for all other covariates (age at diagnosis, disease localization, duration of follow-up, smoking status, etc), a significant association with the rs2395185 variant of the *HLA* gene and steroid-response (P  =  0.024, OR  =  2.07, CI 95% 1.10–3.89), and positive family history (P < 0.01, OR  =  3.68, CI 95% 1.93–7.04) in UC patients was observed. In the CD cohort, the *ZNF365* (rs10761659) SNP was associated with ileal location (L1) compared to patients with colonic (L2) involvement (81.2% vs 72.1%, P  =  0.024, OR  =  1.67, CI 95%, 1.06–2.62). Moreover, colon involvement in CD was significantly more frequent (45%) in patients carrying the minor variant (AA+Aa) of *TNFSF15* (rs4263839) SNP vs a 35% frequency observed in those with only ileal involvement (P  =  0.021, OR  =  1.54, 5% CI, 1.06–2.22). A negative association was found for the minor allele of the *NKX2-3* (rs11190140) gene in UC patients with needs for surgery (P  =  0.038, OR  =  0.64, 95% CI, 0.42–0.97). In CD, the same variant prevailed in patients with smoking habit (P  =  0.018, OR  =  1.46, CI 95%, 1.06–2.00). There was no effect of the other four SNPs (*PTGER4*-rs4613763, *PTPN2*-rs2542151, *IFNG-*rs1558744, *PSMG1*-rs2836878) on all the evaluated sub-phenotypes (data not shown).

**Table 6:** Correlation between risk alleles and clinical characteristics in Crohn's disease (CD) and ulcerative colitis (UC) patients at Multivariate Analysis.

	*CD*		*UC*	
	Adult *n = 728*	Pediatric *n = 342*	Adult *n = 952*	Pediatric *n = 261*
***HLA***rs2395185			***steroid-responsive*** * P = 0.024* *OR 2.07 CI 95% (1.10*–*3.89)*	
			***family history*** * P<0.01* *OR 3.68 CI 95% (1.93*–*7.04)*	
			*Adult P = 0.003* *OR 4.16 CI 95% (1.62*–*10.71)*	*Pediatric P = 0.006* *OR 3.81 CI 95%(1.45*–*9.97)*
***ZNF365***rs10761659	***colon vs ileum*** * P = 0.024* *OR 1.67 CI 95% (1.06*–*2.62)*			
***TNFSF15***rs4263839	***colon vs ileum*** * P = 0.021* *OR 1.54 CI 95% (1.06*–*2.22)*			
***NKX2-3***rs11190140	***smoking*** * P = 0.018* *OR 1.46 CI 95% (1.06*–*2.00)*		***surgery*** * P = 0.038* *OR 0.64 CI 95% (0.42*–*0.97)*	

We asked whether the identified genotype/phenotype associations would differ after stratifying the IBD population in respect to age at diagnosis. For the *HLA*-rs2395185 SNP the association persisted both in the adult- (P  =  0.003) and in the pediatric-onset subset of patients (P  =  0.006) with a positive family history (**Table 6**). Despite the large number of pediatric-onset IBD patients investigated, a trend was found for the genotype/phenotype frequencies of rs10761659, rs4263839, and rs11190140 SNPs, but did not reach statistical significance.

### Gene-gene interactions

The possible interactions of tested variants (rs4613763, rs2395185, rs4263839, rs11190140, rs10761659, rs1558744, rs2542151, and rs2836878) with polymorphisms in the established susceptibility gene *NOD2/CARD15* (the three main polymorphisms rs2066844, rs2066845, and rs2066847: at least 1 variant against wild type) were evaluated. After correction for multiple testing, there was no significant evidence for interaction among the considered SNPs (P > 0.05) (data not shown), thus implying that each gene independently contributes to the disease risk.

## Discussion

Recent GWA studies have enhanced our understanding of the complex genetic architecture of IBD. Most associations appear to be common to both types of IBD, while some genes/loci may be specific to adult- or pediatric-onset, and the factors that determine age of onset are unknown at present.

The major aim of our study was to examine recently described potential association of genes involved in the immune response and inflammation (*PTGER4*, *HLA*, *TNFSF15*, *NKX2-3*, *ZNF365*, *IFNG, PTPN2*, and *PSMG1*) in adult and pediatric-onset IBD in an Italian population.

Using a case-control design, we were able to replicate associations between GWA reported SNPs with CD (*TNFSF15* rs4263839, P  =  7.183×10^−6^; *NKX2-3* rs11190140, P  =  0.003; *ZNF365* rs10761659, P  =  0.007; *PTPN2* rs2542151, P  =  0.037), and UC (*NKX2-3* rs11190140, P  =  4×10^−5^; *ZNF365* rs10761659, P  =  4×10^−5^, *PTGER4* rs4613763, P  =  0.012; *HLA*-*BTNL2* rs2395185, P  =  0.001; *IFNG-IL22-IL26* rs1558744, P  =  0.007; *PSMG1* rs2836878, P  =  0.047).

In addition, our analysis reveals significant association with pediatric UC cohort for six out the eight investigated variants, each of them with a P value < 0.016 (*PTGER4*, *TNFSF15*, *NKX2-3*, *ZNF365*, *IFNG,* and *PSMG1*): this finding suggests that these genes may also be involved in susceptibility to UC pediatric-onset.

To date, no studies on the tested polymorphisms have indicated associations with specific sub-phenotype. We were able to demonstrate, by stepwise logistic regression analysis, a correlation with, *NKX2-3* and need for surgery (P  = 0.038), and with *HLA* and steroid-responsiveness (P  =  0.024), and a positive family history in UC patients. Moreover, significant association in our CD cohort with *TNFSF15* SNP and colonic involvement (P  =  0.021), and with *ZNF365* and ileal location (P  =  0.024) was demonstrated. The clinical significance of these associations, if any, remains to be investigated.

A summary of previously described allelic distributions of SNPs of genes/loci analyzed is depicted in Supplementary Table S1.

Kugathasan *et al* [10] carried out a GWA analysis in a pediatric-onset IBD and identified a significant association of both CD and UC with an intergenic SNP, rs2836878, located on chromosome 21q22 in a small region of linkage disequilibrium that harbors no genes but is close to the *PSMG1* gene. The association was also reiterated by the largest GWA study conducted so far in early-onset IBD (UC: *P = *2.65×10^−9^)[16]. In keeping with previous finding is the contribution of the *PSMG1* locus to disease susceptibility in adult UC [17]. McGovern *et al* [18] confirmed the association after combining data from two new GWA studies and performing a meta-analysis with a published study. A trend for similar associations at rs2836878 variant was observed in CD Canadian cohort but did not achieve statistical significance [19](Table S1). Our results confirm the association in particular with pediatric-onset UC, pinpointing to a significant relevance of the 21q22 for UC. Larger studies that include functional data on the *PSMG1* gene will be required to confirm association at this locus.

A significant association of genetic variants of the *TNFSF15* (*TL1A*) gene on chromosome 9q33 with CD was observed in a large cohort of Japanese patients, in several European cohorts [8], [20], [21], in US Jewish patients [22], in the combined data from the NIDDK IBD Consortium and the WTCCC [3], in Koreans patients [23], and in UC GWA study [7]. The *TNFSF15* is the only gene that has been associated in either Asiatic and European IBD patients [8], [19](Table S1). *TNFSF15* is a member of tumor necrosis factor (TNF) superfamily that binds to death domain receptor 3 (DR3, TNFRSF25) and is expressed in endothelial cells, lymphocytes, plasma cells, monocytes, and dendritic cells [24], [25]. Our analysis confirms the TNFSF15 as a susceptibility locus in CD both in adult and pediatric population (P  =  < 0.001, and P  =  0.008 respectively), and shows an association also with early onset UC (P  =  0.016).

The WTCCC [6] reported on CD cases a novel association involving a cluster of SNPs around the rs10883365 variant on chromosome 10q24.2, which maps within *NKX2-3* (NK2 transcription factor related, locus 3) gene, a member of the NKX family of homeodomain-containing transcription factors. The results of Parkes *et al* [26] supported this findings with an independent set of CD cases and controls of European descent. In addition, a large-scale meta-analysis [3] on CD cohort and replication study on IBD samples [7] found association with rs11190140 polymorphism in complete linkage disequilibrium with the risk allele at the SNP rs10883365 from the WTCCC study (r^2^  =  1.0). In addition, a modest association was also reported with UC in a nonsynonymous SNP scan [27], in GWA scan for UC [17], and in the UC GWA meta-analysis and replication studies [18](Table S1). Similarly, in the present study the rs11190140 variant was associated with an increased risk for both CD and UC (P  =  0.003 and P  =  4×10^−5^ respectively), both in the adult and early-onset cohort. Abnormal expression of NKX2-3 may alter gut migration of antigen-responsive lymphocytes and influence the intestinal inflammatory response. NKX2-3-deficient mice develop splenic and gut-associated lymphoid tissue abnormalities with disordered segregation of T and B cells [28].

A highly attractive candidate gene for IBD, owing to its anti-inflammatory function and involvement in type 1 diabetes susceptibility and rheumatoid arthritis [29], is the *PTPN2* (protein tyrosine phosphatase, non-receptor type 2) located on chromosome 18p11, which encodes for a tyrosine phosphatase expressed in T cells, a negative regulator of inflammation. A novel association at rs2542151 of the *PTPN2* gene and CD was identified [6], replicated [26], [30] and confirmed [3]. In contrast, Franke *et al* [7] observed this association with UC, a finding replicated in the recent GWA on UC [17]. In addition, associations with CD pediatric was also demonstrated [16], [31](Table S1). In the present investigation the association was observed only in adult CD (P  =  0.015) confirming its role as an adult-susceptibility gene.

The UC GWAS in European ancestry samples [9] indentified loci on chromosomes 1p36 and 12q15 where are genes involved in inflammation and immunity, such as *PLA2GIIE* (phospholipase A2, group IIE), *IFNG* (interferon-γ), *IL22* (interleukin-22), and *IL26* (interleukin-26); previous associations were replicated in GWA meta-analysis [18](Table S1). We were able to replicate the association between the rs1558744 SNP on chromosome 12q15 and UC (P  =  0.007), and interestingly after stratifying the cohort with respect to age at diagnosis, the association was observed also in the pediatric-onset patients (adult P  =  0.03; pediatric P  =  0.006).

Several independent genome-wide scans in inflammatory bowel disease have shown evidence of linkage to the MHC region [32], [33], which is characterized by extensive LD blocks (up to 3 Mb) and several genes (250 genes), mainly involved in immune-related functions. Recent genome-wide association studies in UC confirmed the association with the HLA with the maximal association signal at rs2395185, in a region spanning *BTNL2* to *HLA-DQB1* genes [9], [18]. Recently, SNP and the HLA data convincingly show that the main signal is located in a narrow genomic window containing the *HLA-DRB1* gene and strongly suggest that the more common HLA-DRB1*1101 allele plays a primary role in both UC and CD susceptibility [34]. This association to the *DRB1* locus is consistent with other published GWAs in UC [9], [35], [36], also in Japanese population [37]. The same region was identified in the meta-analysis of CD genome-wide association studies [3]. Recent genome-wide association study in early-onset IBD [16] validated this known adult-onset IBD locus in their CD, UC, and IBD dataset, further supporting the importance of this region in IBD risk (Table S1). Allelic and genotype association analysis in our cohort showed that the polymorphism was significantly associated with overall susceptibility to UC (P  =  0.001), and in particular with adult subset (P  =  0.001).

In the study of Libioulle *et al* [38] a region on chromosome 5p13.1 contributing to CD susceptibility was identified. The disease-associated alleles were found to correlate with expression levels of the prostaglandin receptor EP4, which binds prostaglandin E2 (PGE2) and is encoded by *PTGER4.* In the same region the meta-analysis of three GWAS [3] identified rs4613763 as the most strongly associated SNP. No highly significant association of the *PTGER4* region was documented in the studies of the NIDDK [39] and the WTCCC [6]. Recently GWAS showed significant evidence for association also with UC [18] (Table S1). Similarly, our data indicated significant association with adult CD (P  =  0.031), and early-onset UC (P  =  0.002).

In the WTCCC GWA [6], a locus at chromosome 10q21 around rs10761659, a non-coding intergenic SNP mapping 14-kb telomeric to a zinc finger gene known as *ZNF365*, was detected. The locus was replicated both in pediatric CD and UC [16], [31], and adult-onset CD [3], [40] (Table S1). We were able to confirm this association with CD (P  =  0.007), and UC (P  =  4×10^−5^), and after stratifying the cohort with respect to age at diagnosis the association was confirmed in adult CD (P  =  0.002), and either adult (P  =  0.0002), and in pediatric UC patients (P  =  0.005).

In conclusion, our study has confirmed recently described associations, in particular between the *PTGER4*, *HLA*, *TNFSF15*, *NKX2-3*, *ZNF365*, *IFNG, PTPN2*, and *PSMG1* genes and IBD in adults and in some cases for the first time also in children. Furthermore, we were able to identify the influence of the investigated genes on clinical expression and localization on CD and UC patients, but the clinical significance of these associations remains to be investigated and replicated. Further characterization, fine mapping, and functional studies of these genetic regions are needed to discover the pathogenetic role of these newly identified genes/loci.

## Supporting Information

Table S1 Allelic distributions of single nucleotide polymorphisms (SNP) analyzed in the previously published studies and in the current study.
(DOCX)
