# Supplementary material for: Investigation of Multiple Susceptibility Loci for Inflammatory Bowel Disease in an Italian Cohort of Patients
Source: PLoS One. 2011 Jul 27;6(7):e22688. doi: 10.1371/journal.pone.0022688 (PMC3144927; doi:10.1371/journal.pone.0022688)
Supplement: Table S1 — Allelic distributions of single nucleotide polymorphisms (SNP) analyzed in the previously published studies and in the current study. (DOCX) [file pone.0022688.s001.docx]

| **Supplementary Table S1.** | | | | | | | | | | | | | | | | |
| --- | --- | --- | --- | --- | --- | --- | --- | --- | --- | --- | --- | --- | --- | --- | --- | --- |
|  |  |  |  | **Previously published studies** | | | | | |  | **Current study** | | | | | |
| **Chr** | **Gene(s) or locus** | **SNPs analyzed** |  | **Sample size: cases-controls** | **Phenotype associated** | **MAF/RAF control** | **MAF/RAF affected** | **P value** | **OR** |  | **Phenotype associated** | **MAF/RAF control** | **MAF/RAF affected** | **P value** | **OR** | **Ref.** |
| 21q22 | *PSMG1* | rs2836878 |  | 647/4250  317/4250 | CD  UC | 0.27  - | 0.22  0.19 | 0.00026  1.71x10^-5^ | 0.77  0.64 |  | CD  UC | 0.26 | 0.23  0.21 | ns  4.1x10^-3^ | -  0.77 | 10 |
| 21q22 | *PSMG1* | rs2836878 |  | 724/6158 | UC | - | - | 2.65x10^-9^ | - |  |  |  |  |  |  | 16 |
| 21q22 | *PSMG1* | rs2094871 |  | 2361/5417 | UC | - | - | 1.6x10^-6^ | **-** |  |  |  |  |  |  | 17 |
| 21q22 | *PSMG1* | rs2836878 |  | 2693/6791 | UC | - | - | 1.4x10^-8^ | - |  |  |  |  |  |  | 18 |
| 9q32 | *TNFSF15* | rs4263839 |  | 5555/6638 | CD | 0.67 | - | 2.60x10^-10^ | 1.22 |  | CD | 0.69 | 0.76 | 1x10^-6^ | 0.70 | 3 |
| 9q32 | *TNFSF15* | tnfsf15_28 |  | 474/345 | CD | 0.42 | 0.61 | 1.71x10^-14^ | 2.17 |  |  |  |  |  |  | 20 |
| 9q32 | *TNFSF15* | rs7848647 |  | 756/636 | CD | 0.65 | 0.69 | 0.033 | 1.19 |  |  |  |  |  |  | 21 |
| 9q32 | *TNFSF15* | rs7848647 |  | 380/380 | CD | - | - | 4.12x10^-18^ | - |  |  |  |  |  |  | 23 |
| 9q32 | *TNFSF15* | rs6478108 |  | 1748/3000 | CD | - | - | 9.0x10^-5^ | - |  |  |  |  |  |  | 6 |
| 9q32 | *TNFSF15* | rs7869487 |  | 1850/1817 | CD | 0.30 | 0.28 | 0.010 | 0.87 |  |  |  |  |  |  | 7 |
| 10q24 | *NKX2-3* | rs11190140 |  | 5555/6638 | CD | 0.47 | - | 3.06x10^-16^ | 1.20 |  | CD  UC | 0.49 | 0.44  0.42 | 3.5x10^-3^  6x10^-5^ | 1.02  1.31 | 3 |
| 10q24 | *NKX2-3* | rs10883365 |  | 1748/3000 | CD | 0.47 | 0.53 | 1.41x10^-8^ | 1.62 |  |  |  |  |  |  | 6 |
| 10q24 | *NKX2-3* | rs10883365 |  | 1182/2024 | CD | 0.52 | 0.48 | 0.00373 | 1.18 |  |  |  |  |  |  | 26 |
| 10q24 | *NKX2-3* | rs10883365 |  | 1850/1817  1103/1817 | CD  UC | 0.53  0.53 | 0.46  0.49 | 2.44x10^-7^  4.03x10^-5^ | 1.31  1.21 |  |  |  |  |  |  | 7 |
| 10q24 | *NKX2-3* | rs10883365 |  | 1841/1470 | UC | 0.48 | 0.5 | 3.3x10^-4^ | 1.47 |  |  |  |  |  |  | 27 |
| 10q24 | *NKX2-3* | rs6584283 |  | 2361/5417 | UC |  |  | 1.7x10^-7^ | - |  |  |  |  |  |  | 17 |
| 10q24 | *NKX2-3* | rs11190140 |  | 4702/8371 | UC | - | - | 1.1x10^-8^ | - |  |  |  |  |  |  | 18 |
| 18p11 | *PTPN2* | rs2542151 |  | 1748/3000 | CD | 0.16 | 0.20 | 4.56x10^-8^ | 2.01 |  | CD | 0.13 | 0.16 | 0.020 | 1.26 | 6 |
| 18p11 | *PTPN2* | rs2542151 |  | 1182/2024 | CD | 0.19 | 0.17 | 0.0479 | 1.15 |  |  |  |  |  |  | 26 |
| 18p11 | *PTPN2* | rs2542151 |  | 1621/1086 | CD | 0.15 | 0.18 | 7.54x10^-3^ | 1.26 |  |  |  |  |  |  | 30 |
| 18p11 | *PTPN2* | rs2542151 |  | 5555/6638 | CD | 0.15 | - | 5.10x10^-17^ | 1.35 |  |  |  |  |  |  | 3 |
| 18p11 | *PTPN2* | rs2542151 |  | 1850/1817  1103/1817 | CD  UC | 0.15  0.15 | 0.19  0.17 | 1.3x10^-3^  0.01 | 1.33  1.18 |  |  |  |  |  |  | 7 |
| 18p11 | *PTPN2* | rs2542151 |  | 2361/5417 | UC | - | - | 1x10^-3^ | - |  |  |  |  |  |  | 17 |
| 18p11 | *PTPN2* | rs1893217 |  | 1925/7654 | CD | - | - | 4.34x10^-3^ | - |  |  |  |  |  |  | 16 |
| 18p11 | *PTPN2* | rs1893217 |  | 796/784 | CD | 0.14 | 0.19 | 5x10^-3^ | - |  |  |  |  |  |  | 31 |
| 12q15 | *IFNG, IL22, IL26* | rs1558744 |  | 1052/2571 | UC | 0.38 | 0.48 | 5.5x10^-10^ | 1.47 |  | UC | 0.38 | 0.44 | 6x10^-4^ | 1.31 | 9 |
| 12q15 | *IFNG, IL22, IL26* | rs1558744 |  | 4702/8371 | UC |  |  | 4.2X10^-12^ | - |  |  |  |  |  |  | 18 |
| 6p21 | *HLA, BTNL2* | rs2395185 |  | 1052/2571 | UC | 0.33 | 0.24 | 1.4x10^-6^ | 0.72 |  | UC | 0.23 | 0.21 | 4.1x10^-3^ | 0.77 | 9 |
| 6p21 | *HLA, BTNL2* | rs2395185 |  | 2693/6791 | UC | - | - | 8.8x10^-23^ | - |  |  |  |  |  |  | 18 |
| 6p21 | *HLA, BTNL2* | rs9268877 |  | 2361/5417 | UC | - | - | 3.9x10^-23^ | - |  |  |  |  |  |  | 17 |
| 6p21 | *HLA, BTNL2* | rs660895 |  | 905/1465 | UC | 0.23 | 0.17 | 1.5x10^-5^ | - |  |  |  |  |  |  | 35 |
| 6p21 | *HLA, BTNL2* | rs9268877 |  | 1167/777 | UC | 0.44 | 0.53 | 5.23x10^-7^ | 1.51 |  |  |  |  |  |  | 36 |
| 6p21 | *HLA, BTNL2* | rs9263739 |  | 1384/3057 | UC | - | - | 4.15x10^-67^ | 2.73 |  |  |  |  |  |  | 37 |
| 6p21 | *HLA, BTNL2* | rs3763313 |  | 5555/6638 | CD | 0.18 | - | 5.20x10^-9^ | 1.19 |  |  |  |  |  |  | 3 |
| 6p21 | *HLA, BTNL2* | rs660895  rs2395185 |  | 1925/7654  844/7854 | CD  UC |  |  | 1.05x10^-2^  7.40x10^-19^ | -  - |  |  |  |  |  |  | 16 |
| 5p13 | *PTGER4* | rs4613763 |  | 5555/6638 | CD | 0.125 | - | 6.82x10^-27^ | 1.32 |  | CD  UC | 0.08 | 0.11  0.11 | 0.036  8.2x10^-3^ | 1.34  1.44 | 3 |
| 5p13 | *PTGER4* | rs1373692 |  | 1789/1481 | CD | 0.593 | 0.679 | 2.1x10^-12^ | - |  |  |  |  |  |  | 38 |
| 5p13 | *PTGER4* | rs4613763 |  | 4702/8371 | UC |  |  | 4.2x10^-4^ | - |  |  |  |  |  |  | 18 |
| 10q21 | *ZNF365* | rs10761659 |  | 1748/3000 | CD | 0.461 | 0.406 | 2.68x10^-7^ | 1.55 |  | CD  UC | 0.52 | 0.46  0.45 | 1.6x10^-3^  4.2x10^-4^ | 1.28  1.35 | 6 |
| 10q21 | *ZNF365* | rs10995271 |  | 5555/6638 | CD | 0.387 |  | 4.46x10^-20^ | 1.25 |  |  |  |  |  |  | 3 |
| 10q21 | *ZNF365* | rs10995250 |  | 1925/7654  844/7854 | CD  UC |  |  | 1.77x10^7^  4.12x10^-2^ | -  - |  |  |  |  |  |  | 16 |
| 10q21 | *ZNF365* | rs10995271 |  | 347/293 | CD | 0.35 | 0.43 | 1x10-3 | 1.39 |  |  |  |  |  |  | 31 |
| 10q21 | *ZNF365* | rs10995271 |  | 384/312 | CD |  |  | 7.41x10^-7^ | 1.65 |  |  |  |  |  |  | 40 |
| Chr., chromosome; CD, Crohn’s disease; UC, ulcerative colitis; MAF, minor allele frequency; RAF, risk allele frequency; OR, odds ratio | | | | | | | | | | | | | | | | |
